# Kinetics of Phenolic Compounds Modification during Maize Flour Fermentation

**DOI:** 10.3390/molecules26216702

**Published:** 2021-11-05

**Authors:** Oluwafemi Ayodeji Adebo, Ajibola Bamikole Oyedeji, Janet Adeyinka Adebiyi, Chiemela Enyinnaya Chinma, Samson Adeoye Oyeyinka, Oladipupo Odunayo Olatunde, Ezekiel Green, Patrick Berka Njobeh, Kulsum Kondiah

**Affiliations:** 1 Department of Biotechnology and Food Technology, Doornfontein Campus, Faculty of Science, University of Johannesburg, Doornfontein, P.O. Box 17011, Johannesburg 2028, South Africa; janetaadex@gmail.com (J.A.A.); sartf2001@yahoo.com (S.A.O.); egreen@uj.ac.za (E.G.); pnjobeh@uj.ac.za (P.B.N.); 2 Department of Food Science and Technology, Federal University of Technology, P.M.B 65, Minna 920001, Nigeria; chinmachiemela@futminna.edu.ng; 3 Africa Center of Excellence for Mycotoxin and Food Safety, Federal University of Technology, P.M.B 65, Minna 920001, Nigeria; 4 Department of Food and Human Nutritional Sciences, Faculty of Agricultural and Food Sciences, University of Manitoba, Winnipeg, MB R3T 2N2, Canada; oladipupo.olatunde177@gmail.com

**Keywords:** first order, flavonoids, kinetic modelling, phenolic acids, zero order

## Abstract

This study aimed to investigate the kinetics of phenolic compound modification during the fermentation of maize flour at different times. Maize was spontaneously fermented into sourdough at varying times (24, 48, 72, 96, and 120 h) and, at each point, the pH, titratable acidity (TTA), total soluble solids (TSS), phenolic compounds (flavonoids such as apigenin, kaempferol, luteolin, quercetin, and taxifolin) and phenolic acids (caffeic, gallic, ferulic, *p*-coumaric, sinapic, and vanillic acids) were investigated. Three kinetic models (zero-, first-, and second-order equations) were used to determine the kinetics of phenolic modification during the fermentation. Results obtained showed that fermentation significantly reduced pH, with a corresponding increase in TTA and TSS. All the investigated flavonoids were significantly reduced after fermentation, while phenolic acids gradually increased during fermentation. Among the kinetic models adopted, first-order (R^2^ = 0.45–0.96) and zero-order (R^2^ = 0.20–0.82) equations best described the time-dependent modifications of free and bound flavonoids, respectively. On the other hand, first-order (R^2^ = 0.46–0.69) and second-order (R^2^ = 0.005–0.28) equations were best suited to explain the degradation of bound and free phenolic acids, respectively. This study shows that the modification of phenolic compounds during fermentation is compound-specific and that their rates of change may be largely dependent on their forms of existence in the fermented products.

## 1. Introduction

Phenolic compounds are vital constituents of food and secondary metabolites of plants derived from several biosynthetic precursors through the action of shikimate, phenylpropanoid, and pentose phosphate metabolism pathways [1]. These phenolic compounds (also called phenolics) are often encountered in food products, especially those derived from plants and cereals, and are known to exert health benefits such as anticarcinogenic potential, the prevention and counteraction of oxidative stress, chemo-preventive effects, and the reduction of free radical-related cellular damage [2,3,4,5].

Maize (*Zea mays*) is one of the main cereals produced worldwide, providing 30% of food calories to over 4 billion people in the world [6,7]. It is also considered a major staple in 125 developing countries [6,7]. Maize is transformed into other food forms using a wide range of processing techniques, one of them being fermentation. Subsequent fermented maize-based food products play a significant role in the nutrition and diets of inhabitants of developing and underdeveloped countries. The fermentation process not only improves food composition, palatability, sensory properties, digestibility, and nutritional constituents, but also reduces antinutritional properties [8,9,10]. Although fermentation is known to positively impact the phenolic constituents of cereals [4], it is nonetheless important to study the changes in the levels of these beneficial food components during the fermentation process. This is vital, considering the purported roles of these phenolic compounds as health beneficial components in foods.

According to van Boekel [11], kinetic modelling has numerous applications in food, including its use as a tool to understand biochemical modifications in food. Natural (spontaneous) fermentation is a complex process involving interactions between microorganisms, the food substrate, and inherent constituents. It is thus desirable to adopt mathematical equations for kinetic simulations of the phenolic compound modification, as a function of fermentation time, to fully understand the process. To the best of our knowledge, there is a dearth of information on this in the literature. This study aimed to investigate the modifications in the phenolic compounds of maize flour over different fermentation times.

## 2. Results and Discussion

### 2.1. pH, Titratable Acidity (TTA) and Total Soluble Solids (TSS)

The pH and TTA are important biochemical parameters particular to fermented foods. A decrease in pH with a corresponding increase in TTA signifies a progression in microbial activity, i.e., the metabolism of fermenting microorganisms coupled with an accumulation of organic acids produced by fermenting microorganisms. Table 1 shows the pH, TTA, and TSS values of unfermented and fermented maize flour samples (24 to 120 h). The values obtained herein are similar to the values of fermented cereals with a pH between 3.6 and 4.8 [12,13,14]. The pH values of the maize flour declined with an increase in fermentation time, from an initial pH of 6.30 to a final pH of 3.89, after 120 h of fermentation. While a significant (*p* ≤ 0.05) linear decrease was observed till 72 h, a steady pH value of between 3.88–3.90 was observed from 72–120 h. The initial drop in pH could be associated with the actions of fermenting microorganisms causing production and accumulation in organic acids [15], while the insignificant modification in pH between 72–120 h could possibly suggest a saturation of the activity of the fermentation organisms. An inverse relationship between the TTA and pH values was observed, with a significant increase in TTA as fermentation progressed. Both reduced pH and corresponding higher TTA values were also shown to be desirable against pathogenic microorganisms that would not survive the fermented product.

The TSS value, as measured using a refractometer, is an approximate measure of the sugar content. Fermentation of the maize flours also resulted in changes to the soluble solids (Table 1). Generally, TSS values significantly increased over the fermentation periods. However, after 48 and 72 h of fermentation, there were no significant changes in the TSS values, but a 74% increase was recorded after an additional day of fermentation (96 h samples). A further decline of TSS was later recorded after 120 h of fermentation, with a similar pattern being reported by Yousif et al. [16] during the fermentation of sorghum. These modifications in the TSS values suggest sugar metabolism during the fermentation process and further biotransformation of these components at the latter stage of fermentation. The initially observed increase in TSS suggests the release of sugars and carbohydrate-related compounds, and a continued increase with the fermentation time indicates that the rate of sugar utilization by the fermenting microbiota is lower than the rate of sugars released [17]. Similar trends of increase in TSS have also been reported in the fermentation of rice, maize, and sorghum [18,19,20]. Pearson correlation was also used to show the relationship between the pH, TTA, and TSS data. A positive correlation coefficient indicates that a positive increase in the parameter will lead to a positive increase in the other variable. On the contrary, a negative correlation coefficient means that for a positive increase in the parameter, there would be a negative decrease in the other variable. A negative (−0.818) and significant (*p* ≤ 0.05) correlation was recorded between the pH and TSS values, which suggests that a decrease in pH would significantly increase the TSS values. Such an increase could also be attributed to enzymatic activities of the fermenting organisms that hydrolyse complex polysaccharides into simpler ones [21]. A similar negative (−0.936) and significant (*p* ≤ 0.05) correlation between the pH and TTA also alludes to the trend observed for pH and TTA in Table 1. The TTA, TSS, and pH values suggest that, as fermentation progresses, the metabolism of the fermenting microorganisms increases, albeit reaching a saturated point at longer fermentation times.

### 2.2. Free and Bound Flavonoids Contents and Kinetics of Modification

Phenolic compounds exist in both free and bound forms of plant cells, and the free phenolic compounds are generally solvent extractable. Bound phenolics, on the other hand, remain after extraction and adhere to the food matrix after extraction of the free fraction [22]. These bound fractions in food have numerous health potentials, especially for gut health, as well as other benefits documented in the literature [1,4,5,22].

**Figure 1:**
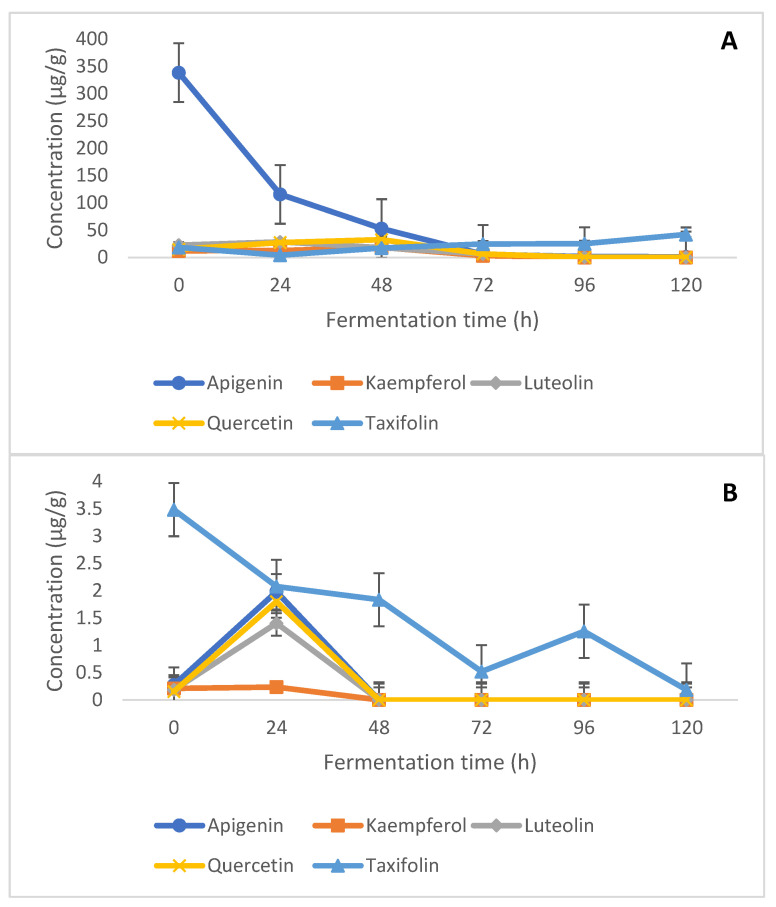
Modification of (**A**) free and (**B**) bound flavonoids in fermented maize flour over time.

Flavonoids are plant secondary metabolites and significant non-nutritive dietary components, as well as a sub-class of phenolic compound groups [4,5,22]. The consumption of these dietary flavonoids from plant-based foods has also been related to the prevention of several chronic diseases, and they are thus seen as vital primary sources for consumption [22]. The trend of modification in the quantified free and bound flavonoid compounds is presented in Figure 1A,B. All five flavonoids (free and bound) investigated in this study were reduced after 120 h of fermentation. This general decrease in the flavonoids (free and bound) suggests that they were rapidly metabolized till they were non-detectable from 48 h onwards for bound apigenin, kaempferol, luteolin, and quercetin (Figure 1B). Apigenin is a naturally occurring flavonoid in yellow maize [23] and, similar to other pigment-related compounds, confers colour to the maize kernel. This compound was significantly higher than other flavonoid compounds in maize, with an initial concentration of 338.42 µg/g to a final concentration of 1.3 µg/g, after 120 h of fermentation (Figure 1A). A similar trend was observed in its bound form from an initial concentration of 0.27 µg/g to it not being detected after 48 h and onwards. Such total degradation of pigment-related compounds has also been reported in fermented sorghum, with the authors ascribing this to the conversion of apigeninidin, and methoxyapigeninidin into novel adducts of deoxyanthocyanidins [24]. Further investigation into the product(s) from such degradation of apigenin in this study should be elucidated in future research and would provide an understanding of the mechanisms involved, as well as any subsequent products formed.

Such decreases in the bound and free flavonoids have been reported during the fermentation of maize and other cereals and can be ascribed to the metabolism of these compounds by fermenting microorganisms, degradation/polymerization of flavonoid compounds into dihydroxyl flavone analogues or derivatives, and the activities of endogenous grain enzymes such as glycosidases, glycosyltransferases, cellulase, tannase, esterase, and hydrolases [4,5,25,26,27]. The noted significant decrease of flavonoid content after longer fermentation periods coincides with the marked decrease in pH values of the fermented samples and is well-reflected in the strong correlation coefficient of these flavonoids with pH. This also suggests that these flavonoids are liable to acidic pH, thus resulting in further hydrolysis at lower pH [28].

**Figure 2:**
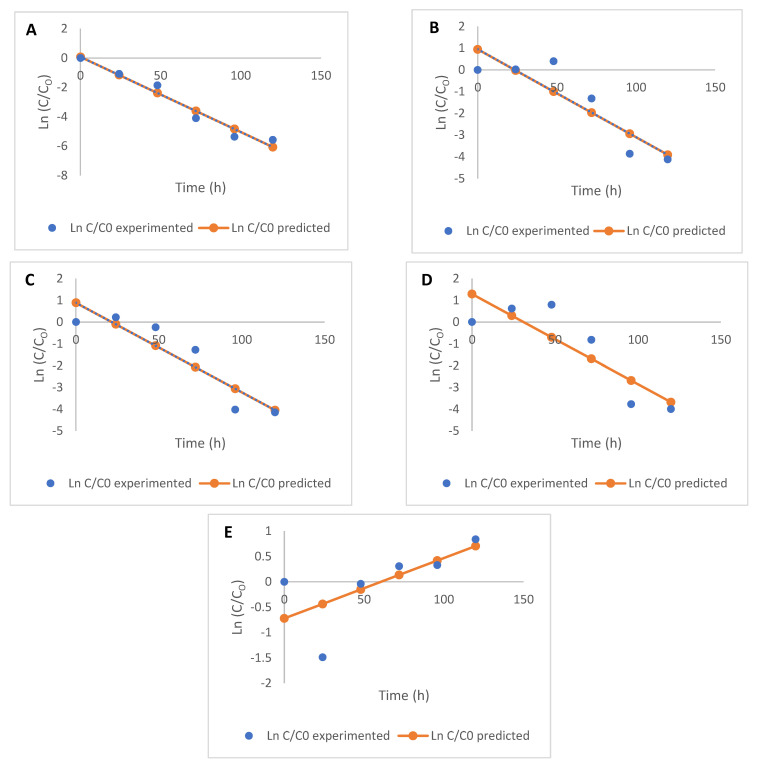
Kinetics (first-order) of free flavonoid degradation during the fermentation of maize flour (**A**) apigenin, (**B**) kaempferol, (**C**) luteolin, (**D**) quercetin, and (**E**) taxifolin.

**Figure 3:**
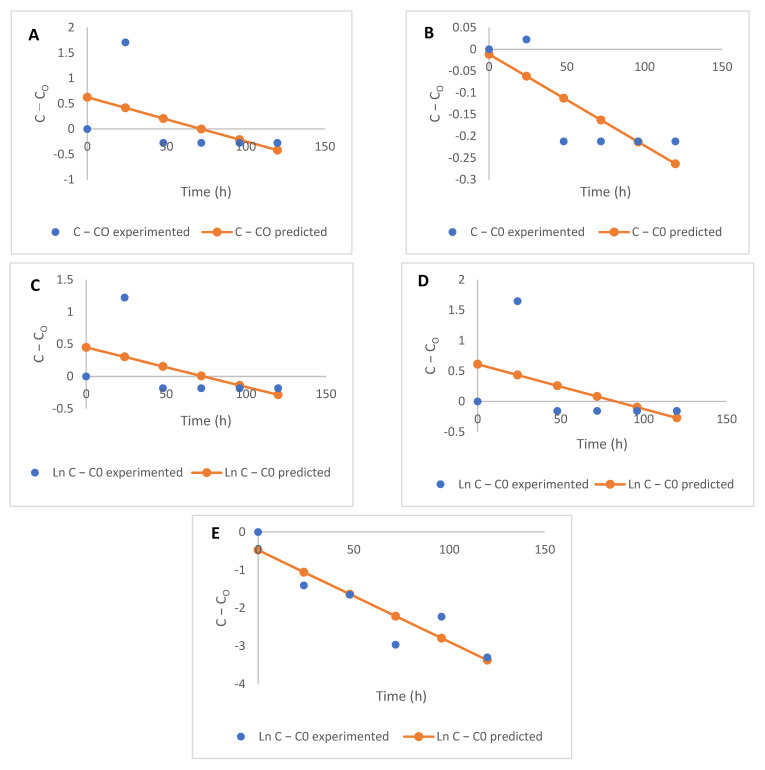
Kinetics (zero-order) of bound flavonoid degradation during the fermentation of maize flour (**A**) apigenin, (**B**) kaempferol, (**C**) luteolin, (**D**) quercetin, and (**E**) taxifolin.

The parameters (rate of degradation, *k*, and the coefficient of determination, R^2^) of each of the models used to describe the kinetic degradation of free and bound flavonoids are presented in Table 2. The first-order kinetics model (R^2^ = 0.45–0.96) best describes the time-dependent degradation of free flavonoids, while the zero-order model (R^2^ = 0.20–0.82) is most suited for describing the kinetics of reduction in bound flavonoids for the fermented maize products, as they both present the highest R^2^ values in each case (Figure 2; Figure 3). The low R^2^ values for the kinetics of some free and bound flavonoids can be attributed to the drastic reduction after 48 h. While there were no other significant changes in the phenolic contents after 48 h, fermentation continued to occur, as reflected in the TTA and TSS values. First-order models have been successfully used to describe the degradation of phenolic compounds in different food systems subjected to various processing operations [29,30,31] and zero-order [32,33]. Low R^2^ values generally obtained for the kinetic models, especially for bound flavonoids, is reflective of the oscillating degradation patterns of these phenolic compounds at the different fermentation times investigated. This can be an effect of various biological reactions, such as decarboxylation, esterification, and hydrolysis occurring simultaneously [4,5,15] and leading to changes at the different fermentation times [29], which could be degradation or synthesis of bioactive components, including phenolic compounds.

The rate constant *k*, for the degradation of free flavonoids, described by the first-order kinetics model, ranged from −2.470 to 0.227 h^−1^, while that of bound flavonoids described by zero-order kinetics ranged from −0.024 to −0.002 h^−1^. The nature of change (degradation or increase) in flavonoids is depicted by the direction of the kinetic curves, hence the different ‘*k*’ values obtained for each model. Rate constants are indications of the speed of progress of biological reactions with time, as authors [34,35] have previously used ‘*k*’ values to predict the progress and compare different biological reactions. Generally, rate constants of zero-order kinetics were the lowest for bound flavonoids compared to the other models used and degradation of free flavonoids also proceeded at lower rates, using first-order kinetics (Table 2).

### 2.3. Free and Bound Phenolic Acids and Kinetics of Modification

**Figure 4:**
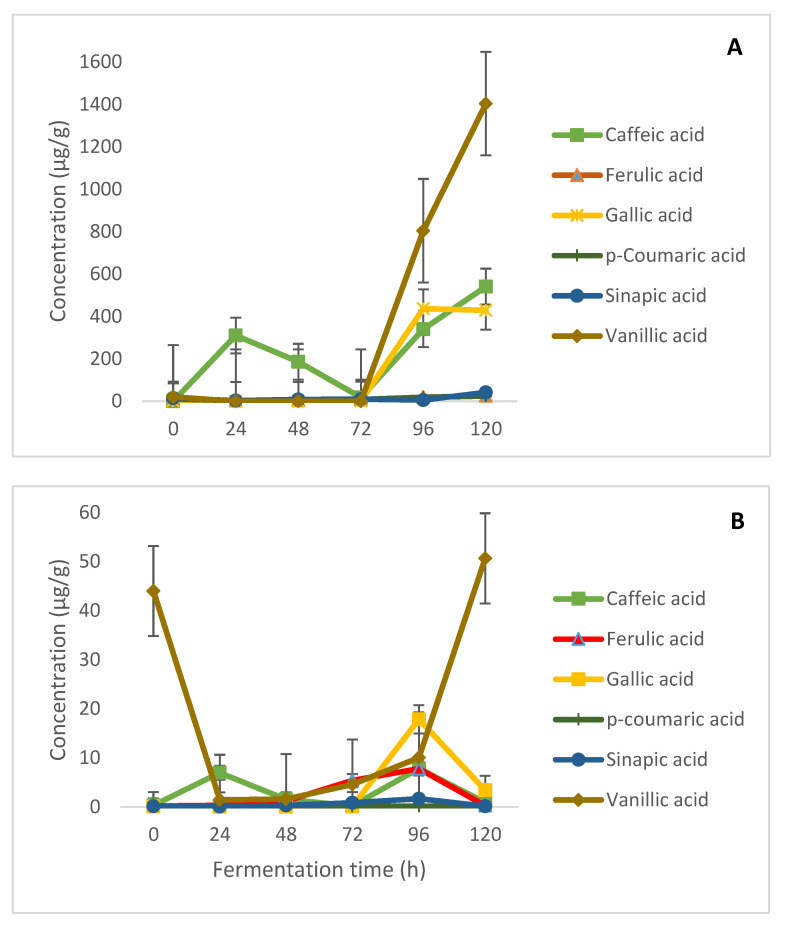
Modification of (**A**) free and (**B**) bound phenolic acids in fermented maize flour over time.

Similar to the flavonoids, all the investigated phenolic acids were present in the initial maize samples prior to fermentation. The free phenolic acid content in the unfermented maize flour ranged from 0.16 µg/g (caffeic acid) to 20.25 µg/g (vanillic acid). The least bound phenolic acid in the unfermented flour was gallic acid (0.10 µg/g), while the highest was vanillic acid (44.01 µg/g). While a consistent decrease trend was observed for the flavonoids, the phenolic acids were generally observed to increase, albeit not in a linear manner (Figure 4A). As posited by several studies, the influence of fermenting microorganisms on the levels of individual phenolics can differ, as it is dependent on the microbial strain and possible genes for phenolic metabolism, as well as fermentation conditions [4,36,37]. Nevertheless, remarkable increases of over 10 000-fold were particularly noted for caffeic, gallic, and vanillic acids in their free forms. Similar increases in phenolic acids after fermentation have been reported in other cereal-based products such as sorghum sourdough [38], fermented barley and oat [39], and spelt wheat *tempe* [40].

Although not as pronounced as in the free form, similar increases were noted for the bound phenolic acids (Figure 4B). The observed general increase in these phenolic acids can be ascribed to the activities of some microbially secreted enzymes that hydrolysed the ester, polymeric, and glycosidic bonds of the phenolic acids, as well as the structural breakdown of cell walls, which invariably resulted in improved bioavailability and extractability [41,42,43]. These mechanisms also contributed to the decrease of the flavonoids (Section 2.2) and efficiently released these phenolic acids from the cell wall of the maize grain. The remarkable decrease in apigenin noted in this study may also have contributed to the increase in phenolic acids. Vernhet et al. [44] also reported this observation in their study on the fermentation of red musts, noting that the interconversion between phenolic compounds and the breakdown of pigment-related compounds could contribute to an increase in phenolic acids.

Contrary to the kinetics of bound flavonoids, the first-order kinetic model more appropriately described the time-dependent changes in bound phenolic acids. Both first- and second-order kinetics better described the effect of fermentation on free phenolics with comparable R^2^ and *k* values, as compared to zero-order kinetics (Table 2). However, second-order kinetics were preferable in describing the changes in free phenolic acids, with the highest reaction rate constants of the three models used. The R^2^ values obtained for bound phenolic acids using first-order kinetics (0.02–0.59) were higher than those obtained when zero-order (0.004–0.28) and second-order (0.005–0.28) were used. The release of bound phenolics from fermented maize products was depicted to have proceeded at faster rates for all bound phenolic acids by zero- and second-order kinetic models, evident by the higher *k* values compared to first-order kinetics which produced the lowest *k* values. However, the higher coefficients of determination (R^2^) produced by the first-order kinetics showed that the model better described the changes in bound phenolic acids, despite lower rates than were described by the zero- and second-order kinetics—hence the choice of first-order kinetics.

**Figure 5:**
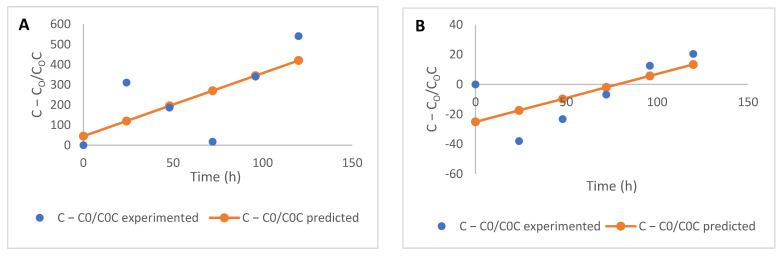

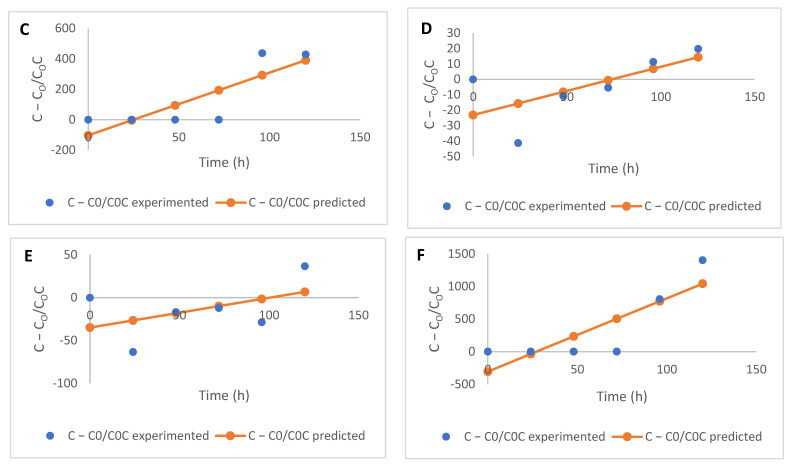
Kinetics (second-order) of free phenolic acid degradation during the fermentation of maize flour (**A**) caffeic acid, (**B**) ferulic acid, (**C**) gallic acid, (**D**) *p*-coumaric acid, (**E**) sinapic acid, and (**F**) vanillic acid.

**Figure 6:**
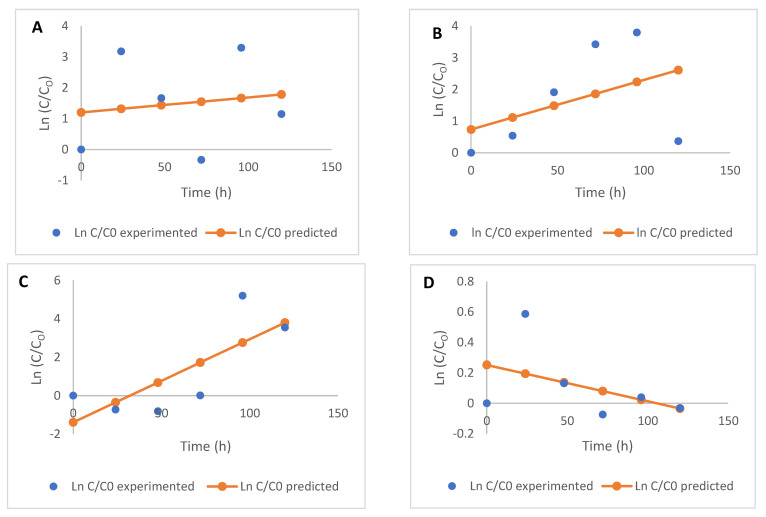

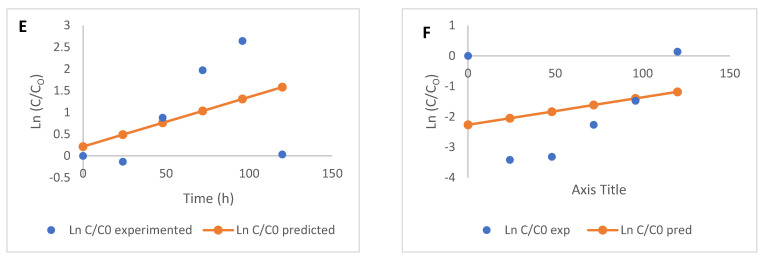
Kinetics (first-order) of bound phenolic acid degradation during the fermentation of maize flour (**A**) caffeic acid, (**B**) ferulic acid, (**C**) gallic acid, (**D**) *p*-coumaric acid, (**E**) sinapic acid, and (**F**) vanillic acid.

For free phenolic acids, first-order rate constant ‘*k*’ ranged between 0.012–11.110 h^−1^ (R^2^ = 0.46–0.69), while for zero- and second-order kinetics, *k* ranged between −0.038–0.146 mgh^−1^ (R^2^ = 0.44–0.70) and 0.312–11.227 mgh^−1^ g^−1^ h^−1^ (R^2^ = 0.46–0.70), respectively. Slight similarities in *k* values for the first- and second-order reaction for free phenolic compounds suggests that the reactions proceeded at similar rates for both models. Also, rate constants showed that changes in free phenolic acids proceeded at faster rates, as compared to bound phenolic compounds (Table 2)—a trend also shown by the kinetic model curves (Figure 5 and Figure 6). These faster rates could be because of their bioavailability as free phenolics in the fermenting matrices. The degradation of free vanillic acid had the highest ‘*k*’ and R^2^ values, as described by the first- and second-order kinetics. De Beer et al. [29] had previously used zero-, first-, and second-order kinetics to describe the rate of changes in the phenolic compounds of rooibos tea fermented at different temperatures, and found that first-order kinetics was the most suitable model for describing the degradation of these compounds.

### 2.4. Principal Component Analysis

**Figure 7:**
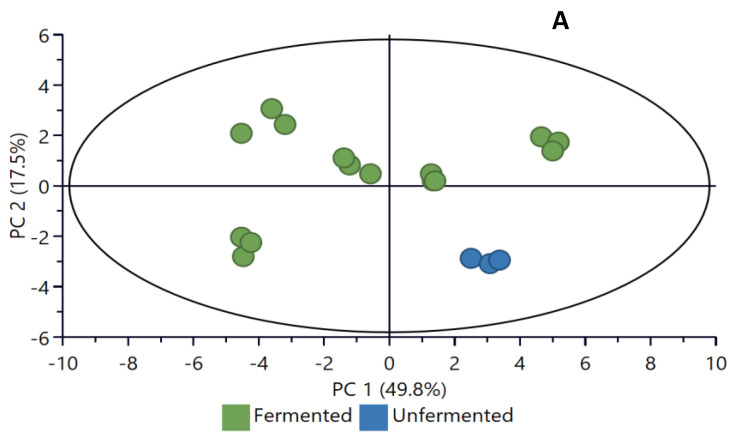

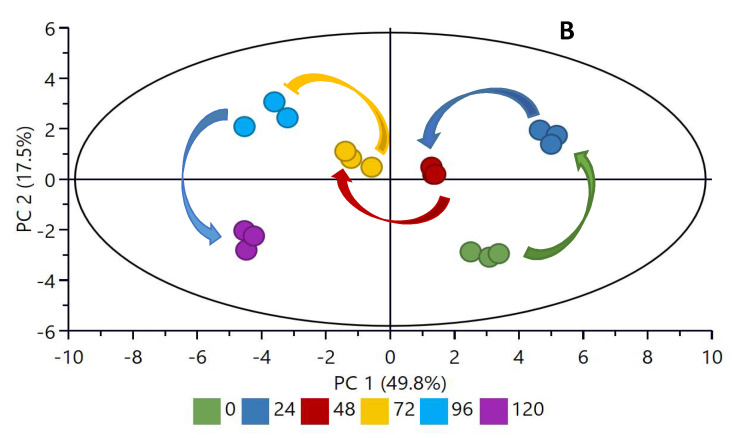
Exploratory data analysis with PCA. (**A**) Relationship of unfermented and fermented samples; (**B**) relationship of samples fermented at different times.

The PCA was used to explore the distribution and highlight natural groupings, as well as the trends and relationship of the parameters investigated in the unfermented and fermented maize flour samples. The first two principal components (PCs), PC1 and PC2, explained 49.8% and 17.5%, respectively, of the variation (total of 67.3%). The unfermented maize flour (0 h) on the bottom right quadrant showed a clear separation from the fermented samples (grouped in other parts on the PC plot (Figure 7A). A further classification based on the fermentation periods (Figure 7B) showed the different groupings as a result of the fermentation time. Clusters of the 24 and 48 h fermented samples moved on the right of the PC1 quadrant, while clusters for the later stages (72–120 h) of fermentation moved to the left part of the PC1 quadrant. The observed separation and clusters of the parameters investigated in the maize samples are suggestive of differential changes during fermentation and correlate with the results of the pH, TTA, and TSS (Table 1), as well as the phenolic compounds (Figure 1; Figure 4).

## 3. Materials and Methods

Yellow maize (*Zea mays*) grains (variety IMP50BR) were obtained from the Agricultural Research Council (Grain Crops, Potchefstroom, South Africa). The grains were milled using a Perten Laboratory Mill 3310 (Perten Instruments AB, Helsinki, Finland) and passed through a 0.5 mm aperture size sieve (Analysette 3 Spartan, Fritsch, Germany) to obtain the flour.

### 3.1. Fermentation

Maize flour was processed into sourdough by mixing it with sterile distilled water (1:1, *v/w*). The mixture was then incubated (Incotherm, Labotec, Johannesburg, South Africa) at 37 °C and fermentation was separately done for 24, 48, 72, 96, and 120 h. For each time, the fermentation process was performed in triplicates.

### 3.2. Total Soluble Solids (TSS), pH, and Titratable Acidity (TTA)

A refractometer (HI 96801, HANNA Instruments, Inc., Cluj-Napoca, Romania) was used to measure the total soluble solids of samples, while an initially calibrated pH meter (pH 510, Eutech Pte Ltd., Taus, Singapore) was used to obtain their pH values. For titratable acidity (TTA), the method described by Aguilar et al. [45] was used. It involved mixing 10 g of each sample in 90 mL of distilled water, with the mixture being allowed to rest to obtain the supernatant. Thereafter, the supernatant was titrated against 0.1 M NaOH (pH 8.3).

### 3.3. Extraction and Quantification of Free and Bound Phenolic Compounds

Prior to quantification, phenolic compounds were extracted using the modified methods of Xiang et al. [46] and Ravisankar et al. [47]. A 0.25 g of the raw ground and fermented maize samples, as well as 2.5 mL of acidified methanol (1% HCl in 80% aqueous methanol), were added. The mixture was sonicated in an ultrasonic bath (AU 220, Argo Lab, Carpi, Italy) for 1 h at 4 °C, followed by centrifugation (Eppendorf 5702R, Merck, Germiston, South Africa) at 2100× *g* at 4 °C for 10 min. The supernatant (free phenolics) was evaporated using a vacuum concentrator (Eppendorf Concentrator Plus, Analytical Solutions, Johannesburg, South Africa) and the dried extract was reconstituted with 1 mL 50% liquid chromatographic-grade methanol (Merck, Johannesburg, South Africa). The residue earlier obtained was then hydrolysed (for 30 min at 60 °C) with 2.5 mL of 2 M NaOH and 2.5 mL of ethyl acetate. A similar process of sonication and centrifugation was performed. The supernatant (bound phenolics) was evaporated using a vacuum concentrator, and the dried extract was reconstituted with 1 mL 50% liquid chromatographic-grade methanol.

Quantification of the free and bound phenolics was performed on an ultra-high pressure liquid chromatography (UHPLC) system (Shimadzu, Kyoto, Japan) equipped with a degassing unit (DGU-403), binary pumps (LC-40B XR), solvent delivery module (LC-40B XR), auto-sampler (SIL-40C XR), column oven (CTO-40C), and a diode array detector (DAD) (SPD-M40). A two (2) µL extract was injected into the system, and separation (Supplementary Materials Figure S1) was carried out on a Raptor C18 column (2.7 μm × 100 mm × 2.1 mm ID, Restek, Bellefonte, USA) at an oven temperature of 40 °C. Mobile phase A consisted of 1% formic acid in Milli-Q water and B was 1% formic acid in a mixture of 50% methanol and acetonitrile, with the total run time being 15 min. Quantification of each phenolic compound, including the apigenin, caffeic acid, gallic acid, ferulic acid, kaempferol, luteolin, *p*-coumaric acid, quercetin, sinapic acid, taxifolin, and vanillic acid (Sigma Aldrich, Johannesburg, South Africa), was executed through extrapolation from the calibration curves of analytical standards at different concentrations (2.5, 5, 10, 20, 40, 80, and 160 µg/mL) (Supplementary Materials Figure S2). Table 3 shows the retention times and wavelengths of each phenolic compound.

### 3.4. Kinetic Modelling

To understand the kinetics of changes in the phenolic compounds in this study, zero-, first-, and second-order mathematical models were adopted. The use of these models has been widely adopted to describe reaction processes in biological systems, especially foods [14]. Linearized forms of these models’ Equations (1)–(3) were used to determine the kinetic parameters using Datafit 9.1 software (Oakdale Engineering, Oakdale, PA, USA).

(1) $$\mathrm{Zero}\;\mathrm{order}:C-C_{0}=kt$$

(2) $$\mathrm{First}\;\mathrm{order}:ln\frac{C}{C_{0}}=kt$$

(3) $$\mathrm{Second}\;\mathrm{order}:\frac{C-C_{0}}{C_{0}C}=kt$$

where *C* is the concentration of phenolic acids at a specific experimental time (*t*), *C*_0_ is the initial concentration at 0 h, *k* is the rate constant, and *t* is the time.

### 3.5. Statistical Analysis

All analyses were done in triplicates and the differences between the means were established using analysis of variance, followed by Tukey’s test (SPSS 22, IBM, Armonk, NY, USA). Variances at a 5% confidence level were considered to be statistically different. Pearson’s correlation test and the principal component analysis (PCA) (SIMCA 16, Umetrics, Umea, Sweden) were also conducted on the investigated parameters.

## 4. Conclusions

Fermentation of maize over a period of 120 h showed modifications in both the biochemical parameters (pH, TTA, and TSS) and the phenolic compounds (flavonoids and phenolic acids). While the flavonoids followed a general decrease, there was an increase in the phenolic acids, suggestive of different metabolism and modification routes of the phenolic compounds. Although higher levels of both flavonoids and phenolic acids are desirable due to the health benefits they confer, the general decrease in the flavonoids might have led to other equally bioactive monomers of significance. Among the three different kinetic models used, first-order was notably adequate for bound flavonoids and phenolic acids, with zero-order and second-order effectively describing the time-dependent degradation of other phenolic compound groups. Trends of such modifications could also be important in deciding fermentation times during the processing of maize-based products. However, further studies are required on the elucidation of the degraded phenolic compounds using liquid chromatography coupled with mass spectrometry systems, perhaps in combination with metabolomic techniques, to understand the mechanisms and metabolism of degradation and metabolic products. Equally important is the isolation of the fermenting microorganisms and in vitro investigation into the specific phenolic metabolism of the respective microorganisms.

## Figures and Tables

**Table 1:** pH, TTA, and TSS values of maize sourdough over different fermentation periods.

Fermentation Time (h)	pH	TTA (g/kg)	TSS (°Brix)
0	6.30 ^c^ ± 0.14	0.27 ^a^ ± 0.06	0.20 ^a^ ± 0
24	5.21 ^b^ ± 0.11	0.57 ^b^ ± 0.03	0.20 ^a^ ± 0.10
48	4.01 ^a^ ± 0.03	1.05 ^c^ ± 0.18	0.53 ^ab^ ± 0.25
72	3.88 ^a^ ± 0.11	1.65 ^d^ ± 0.22	0.50 ^ab^ ± 0.10
96	3.90 ^a^ ± 0.04	1.45 ^d^ ± 0.05	0.87 ^c^ ± 0.15
120	3.89 ^a^ ± 0.08	1.53 ^d^ ± 0.06	0.63 ^bc^ ± 0.06

TTA—titratable acidity; TSS—total soluble solids. Values represent mean ± standard deviation of triplicate measurements. Values with differing letters within a row are significantly different at *p* *≤* 0.05.

**Table 2:** Kinetics of phenolic degradation using zero-, first-, and second-order reactions.

	Free Phenolic Compounds						Bound Phenolic Compounds					
	Zero Order		First Order		Second Order		Zero Order		First Order		Second Order	
	k (mgh^−1^)	R^2^	k (h^−1^)	R^2^	k (mgh^−1^ g^−1^ h^−1^)	R^2^	k (mgh^−1^)	R^2^	k (h^−1^)	R^2^	k (mgh^−1^ g^−1^)	R^2^
**Flavonoids**												
Apigenin	−0.015	0.71	−2.470	0.96	−0.007	0.82	−0.009	0.24	−0.007	0.15	−0.007	0.15
Kaempferol	−0.040	0.62	−0.134	0.79	−6.655	0.74	−0.002	0.66	−0.0004	0.15	−0.0002	0.15
Luteolin	−0.041	0.85	−0.248	0.84	−13.268	0.73	−0.006	0.24	−0.007	0.15	−0.005	0.15
Quercetin	−0.214	0.48	−0.041	0.74	−0.214	0.48	−0.007	0.20	−0.009	0.15	−0.006	0.15
Taxifolin	0.012	0.66	0.227	0.45	0.538	0.40	−0.024	0.82	−0.021	0.76	−0.432	0.60
**Phenolic Acids**												
Caffeic Acid	0.046	0.46	3.125	0.44	3.125	0.46	−0.005	0.004	−0.005	0.02	−0.006	0.005
Ferulic Acid	0.146	0.44	0.012	0.53	0.321	0.43	0.032	0.20	−0.016	0.18	0.033	0.20
Gallic Acid	0.056	0.68	4.107	0.68	4.112	0.68	0.083	0.28	0.043	0.59	0.085	0.28
*p*-Coumaric Acid	0.012	0.60	0.139	0.47	0.312	0.44	−0.0004	0.19	−0.002	0.19	−0.0007	0.19
Sinapic Acid	0.009	0.30	0.174	0.20	0.384	0.22	0.006	0.20	0.0114	0.19	0.007	0.20
Vanillic Acid	−0.038	0.69	11.110	0.70	11.227	0.70	0.074	0.02	0.009	0.07	5.160	0.15

**Table 3:** Retention times and wavelengths of the quantified phenolic compounds.

Compound	Retention Time (min)	Wavelength (nm)
**Flavonoids**		
Apigenin	11.324	336
Kaempferol	11.347	366
Luteolin	10.670	348
Quercetin	10.597	371
Taxifolin	9.300	290
**Phenolic acids**		
Caffeic acid	8.597	323
Ferulic acid	9.447	322
Gallic acid	8.528	271
*p*-Coumaric acid	9.348	309
Sinapic acid	9.016	319
Vanillic acid	8.713	261

## Data Availability

Not applicable.

## Supplementary Materials

The following are available online, Figure S1: An example of chromatographic separation of analytes in the sample (with peaks of each phenolic compound); Figure S2: Standard calibration curves and R^2^ values for (A) apigenin, (B) kaempferol, (C) luteolin, (D) quercetin, (E) taxifolin, (F) caffeic acid, (G) ferulic acid, (G) gallic acid, (H) p-coumaric acid, (I) sinapic acid and (J) vanillic acid.

## Sample Availability

Samples studied herein are available on reasonable request from the authors.

## References

[1] Li W., Beta T., Ramawat K., Mérillon J.M. (2013). Food sources of phenolics compounds. Natural Products.

[2] Olatunde O.O., Benjakul S., Vongkamjan K. (2018). Antioxidant and antibacterial properties of guava leaf extracts as affected by solvents used for prior dechlorophyllization. J. Food Biochem..

[3] Bonta R.K. (2019). Dietary phenolic acids and flavonoids as potential anti-cancer agents: Current state of the art and future perspectives. Anti-Cancer Agents Med. Chem.

[4] Adebo O.A., Medina-Meza I. (2020). Impact of fermentation on the phenolic compounds and antioxidant activity of whole cereal grains: A mini review. Molecules.

[5] Leonard W., Zhang P., Ying D., Adhikari B., Fang Z. (2021). Fermentation transforms the phenolic profiles and bioactivities of plant-based foods. Biotechnol. Adv..

[6] FAOSTAT Crop Yields. http://www.fao.org/faostat/en/#data/QC.

[7] Nyirenda H., Mwangomba W., Nyirenda E.M. (2021). Delving into possible missing links for attainment of food security in Central Malawi: Farmers’ perceptions and long term dynamics in maize (*Zea mays* L.) production. Heliyon.

[8] Adebo O.A., Njobeh P.B., Adeboye A.S., Adebiyi J.A., Sobowale S.S., Ogundele O.M., Kayitesi E., Panda S., Shetty P. (2018). Advances in fermentation technology for novel food products. Innovations in Technologies for Fermented Food and Beverage Industries.

[9] Adebiyi J.A., Obadina A.O., Adebo O.A., Kayitesi E. (2018). Fermented and malted millet products in Africa: Expedition from traditional/ethnic foods to industrial value added products. Crit Rev. Food Sci. Nutr..

[10] Adebo O.A. (2020). African sorghum-based fermented foods: Past, current and future prospects. Nutrients.

[11] van Boekel M.A.J.S. (2020). On the pros and cons of Bayesian kinetic modeling in food science. Trends Food Sci. Technol..

[12] Sekwati-Monang B., Gänzle M.G. (2011). Microbiological and chemical characterization of *ting*, a sorghum-based sourdough product from Botswana. Int. J. Food Microbiol..

[13] Edema M.O. (2011). A modified sourdough procedure for non-wheat bread from maize meal. Food Bioproc. Technol..

[14] Decimo M., Quattrini M., Ricci G., Fortina M.G., Brasca M., Silvetti T., Manini F., Erba D., Criscuoli F., Casiraghi M.C. (2017). Evaluation of microbial consortia and chemical changes in spontaneous maize bran fermentation. AMB Express.

[15] Adebo O.A., Njobeh P.B., Adebiyi J.A., Kayitesi E. (2018). Co-influence of fermentation time and temperature on physicochemical properties, bioactive components and microstructure of *ting* (a Southern African food) from whole grain sorghum. Food Biosci..

[16] Yousif N.E., El-Tinay A.H. (2001). Effect of fermentation on sorghum protein fractions and in vitro protein digestibility. Plant. Foods Human Nutr..

[17] Chai K.F., Adzahan N.M., Karim R., Rukayadi Y., Ghazali H.M. (2018). Effects of fermentation time and turning intervals on the physicochemical properties of rambutan (*Nephelium lappaceum* L.) fruit sweatings. Sains Malays..

[18] Padhye V.W., Salunkhe D.K. (1979). Biochemical studies on black gram (*Phaseolus mungo* L.) III. Fermentation of black gram and rice blend and its influence on the in vitro protein digestibilities of proteins. J. Food Biochem..

[19] El Tinay A.H., El Mahdi Z.M., El Soubki A. (1985). Supplementation of fermented sorghum *kisra* bread with legume protein. J. Food Technol..

[20] Yousif N.E., El Tinay A.H. (2000). Effect of fermentation on protein fractions and in vitro protein digestibility of maize. Food Chem..

[21] Hansen C.E., del Olmo M., Burri C. (1998). Enzyme activities in cocoa beans during fermentation. J. Sci Food Agric..

[22] Pérez-Jiménez J., Torres J.L. (2011). Analysis of nonextractable phenolic compounds in foods: The current state of the art. J. Sci Food Agric..

[23] Righini S., Rodriguez E.J., Berosich C., Grotewold E., Casati P., Ferreyra M.L.F. (2019). Apigenin produced by maize flavone synthase I and II protects plants against UV-B-induced damage. Plant. Cell Environ..

[24] Bai Y., Findlay B., Maldonado A.F.S., Schieber A., Vederas J.C., Ganzle M.G. (2014). Novel pyrano and vinylphenol adducts of deoxyanthocyanidins in sorghum sourdough. J. Agric. Food Chem..

[25] Huynh N.T., Van Camp J., Smagghe G., Raes K. (2014). Improved release and metabolism of flavonoids by steered fermentation processes: A review. Int. J. Mol. Sci..

[26] Ali F., Rahul, Naz F., Jyoti S., Siddique Y.H. (2017). Health functionality of apigenin: A review. Int. J. Food Prop..

[27] Oladeji B.S., Akanbi C.T., Gbadamosi S.O. (2017). Effects of fermentation on antioxidant properties of flours of a normal endosperm and quality protein maize varieties. J. Food Measur. Character..

[28] Yang L., Allred K., Dykes L., Allred C., Awika J.M. (2015). Enhanced action of apigenin and naringenin combination on estrogen receptor activation in non-malignant colonocytes: Implications on sorghum-derived phytoestrogens. Food Funct..

[29] De Beer D., Tobin J., Walczak B., Van der Rijst M., Joubert E. (2019). Phenolic composition of rooibos changes during simulated fermentation: Effect of endogenous enzymes and fermentation temperature on reaction kinetics. Food Res. Int..

[30] Karaaslan M., Yilmaz F.M., Cesur Ö., Vardin H., Ikinci A., Dalgiç A.C. (2014). Drying kinetics and thermal degradation of phenolic compounds and anthocyanins in pomegranate arils dried under vacuum conditions. Int. J. Food Sci. Technol..

[31] Turturică M., Stănciuc N., Bahrim G., Râpeanu G. (2016). Effect of thermal treatment on phenolic compounds from plum (*Prunus domestica*) extracts–A kinetic study. J. Food Eng..

[32] Rękas A., Ścibisz I., Siger A., Wroniak M. (2017). The effect of microwave pretreatment of seeds on the stability and degradation kinetics of phenolic compounds in rapeseed oil during long-term storage. Food Chem..

[33] Ali A., Chong C.H., Mah S.H., Abdullah L.C., Choong T.S.Y., Chua B.L. (2018). Impact of storage conditions on the stability of predominant phenolic constituents and antioxidant activity of dried piper betle extracts. Molecules.

[34] Oyedeji A.B., Sobukola O.P., Henshaw F.O., Adegunwa M.O., Sanni L.O., Tomlins K.I. (2016). Kinetics of mass transfer during deep fat frying of yellow fleshed cassava root slices. Heat Mass Transf..

[35] Oyedeji A.B., Sobukola O.A., Green E., Adebo O.A. (2021). Physical properties and water absorption kinetics of three varieties of Mucuna beans. Sci. Rep..

[36] Moore J., Cheng Z., Hao J., Guo G., Liu J.-G., Lin C., Yu L. (2007). Effects of solid-state yeast treatment on the antioxidant properties and protein and fiber compositions of common hard wheat bran. J. Agric. Food Chem..

[37] Starzyńska-Janiszewska A., Stodolaka B., Gómez- Caravaca A.M., Mickowska B., Martin-Garcia B., Byczyńskia L. (2019). Mould starter selection for extended solid-state fermentation of quinoa. LWT Food Sci. Technol..

[38] Svensson L., Sekwati-Monang B., Lutz D.L., Schieber A., Gänzle M.G. (2010). Phenolic acids and flavonoids in nonfermented and fermented red sorghum (*Sorghum bicolor* (L.) Moench). J. Agric. Food Chem..

[39] Hole A.S., Rud I., Grimmer S., Sigl S., Narvhus J., Sahlstrøm S. (2012). Improved bioavailability of dietary phenolic acids in whole grain barley and oat groat following fermentation with probiotic *Lactobacillus acidophilus*, *Lactobacillus johnsonii*, and *Lactobacillus reuteri*. J. Agric. Food Chem..

[40] Starzyńska-Janiszewska A., Stodolak B., Socha R., Mickowska B., Wywrocka-Gurgul A. (2019). Spelt wheat *tempe* as a value-added whole-grain food product. LWT Food Sci. Technol..

[41] Zhang L., Gao W., Chen X., Wang H. (2014). The effect of bioprocessing on the phenolic acid composition and antioxidant activity of wheat bran. Cereal Chem..

[42] Kadiri O. (2017). A review on the status of the phenolic compounds and antioxidant capacity of the flour: Effects of cereal processing. Int. J. Food Prop..

[43] Skrajda-Brdak M., Konopka I., Tańska M., Czaplicki S. (2019). Changes in the content of free phenolic acids and antioxidative capacity of wholemeal bread in relation to cereal species and fermentation type. Eur. Food Res. Technol..

[44] Vernhet A., Carrillo S., Rattier A., Verbaere A., Cheynier V., Nguela J.M. (2020). Fate of anthocyanins and proanthocyanidins during the alcoholic fermentation of thermovinified red musts by different *Saccharomyces cerevisiae* strains. J. Agric. Food Chem..

[45] Aguilar N., Albanell E., Miñarro B., Capellas M. (2016). Chestnut flour sourdough for gluten-free bread making. Eur. Food Res. Technol..

[46] Xiang J., Apea-Bah F.B., Ndolo V.U., Katundu M.C., Beta T. (2019). Profile of phenolic compounds and antioxidant activity of finger millet varieties. Food Chem..

[47] Ravisankar S., Abegaz K., Awika J.M. (2018). Structural profile of soluble and bound phenolic compounds in teff (*Eragrostis tef*) reveals abundance of distinctly different flavones in white and brown varieties. Food Chem..

