# Supplementary material for: Kinetics of Phenolic Compounds Modification during Maize Flour Fermentation
Source: Molecules. 2021 Nov 5;26(21):6702. doi: 10.3390/molecules26216702 (PMC8587012; doi:10.3390/molecules26216702)
Supplement: Supplementary file 1 [file molecules-26-06702-s001.zip › molecules-1396107-supplementary.pdf]

molecules

MDPI

Article

Kinetics of Phenolic Compounds Modification During Maize Flour Fermentation-Supplementary Figures

Oluwafemi Ayodeji Adebo [1,*], Ajibola Bamikole Oyedeji [1,*], Janet Adeyinka Adebiyi [1], Chiemela Enyinnaya Chinma [2,3], Samson Adeoye Oyeyinka [1], Oladipupo Odunayo Olatunde [4], Ezekiel Green [1], Patrick Berka Njobeh [1] and Kulsum Kondiah [1,*]

1 Department of Biotechnology and Food Technology, Faculty of Science, University of Johannesburg (Doornfontein Campus), P. O. Box 17011, Doornfontein 2028, Johannesburg, South Africa; janetaadex@gmail.com (J.A.A); sartf2001@yahoo.com (S.A.O); egreen@uj.ac.za (E.G); pnjobeh@uj.ac.za (P.B.N.)
2 Department of Food Science and Technology, Federal University of Technology Minna, Nigeria; chinmachiemela@futminna.edu.ng
3 Africa Center of Excellence for Mycotoxin and Food Safety, Federal University of Technology Minna, Nigeria
4 Department of Food and Human Nutritional Sciences, Faculty of Agricultural and Food Sciences, University of Manitoba, Winnipeg, MB, R3T 2N2, Canada; oladipupo.olatunde177@gmail.com
* Correspondence: oadebo@uj.ac.za (O.A.A); jibanky2@gmail.com (A.B.O.); kulsumk@uj.ac.za (K.K.); Tel.: +27115596261 (O.A.A.); Tel.: +27744113712 (A.B.O.); Tel.: +27115596915 (K.K.)

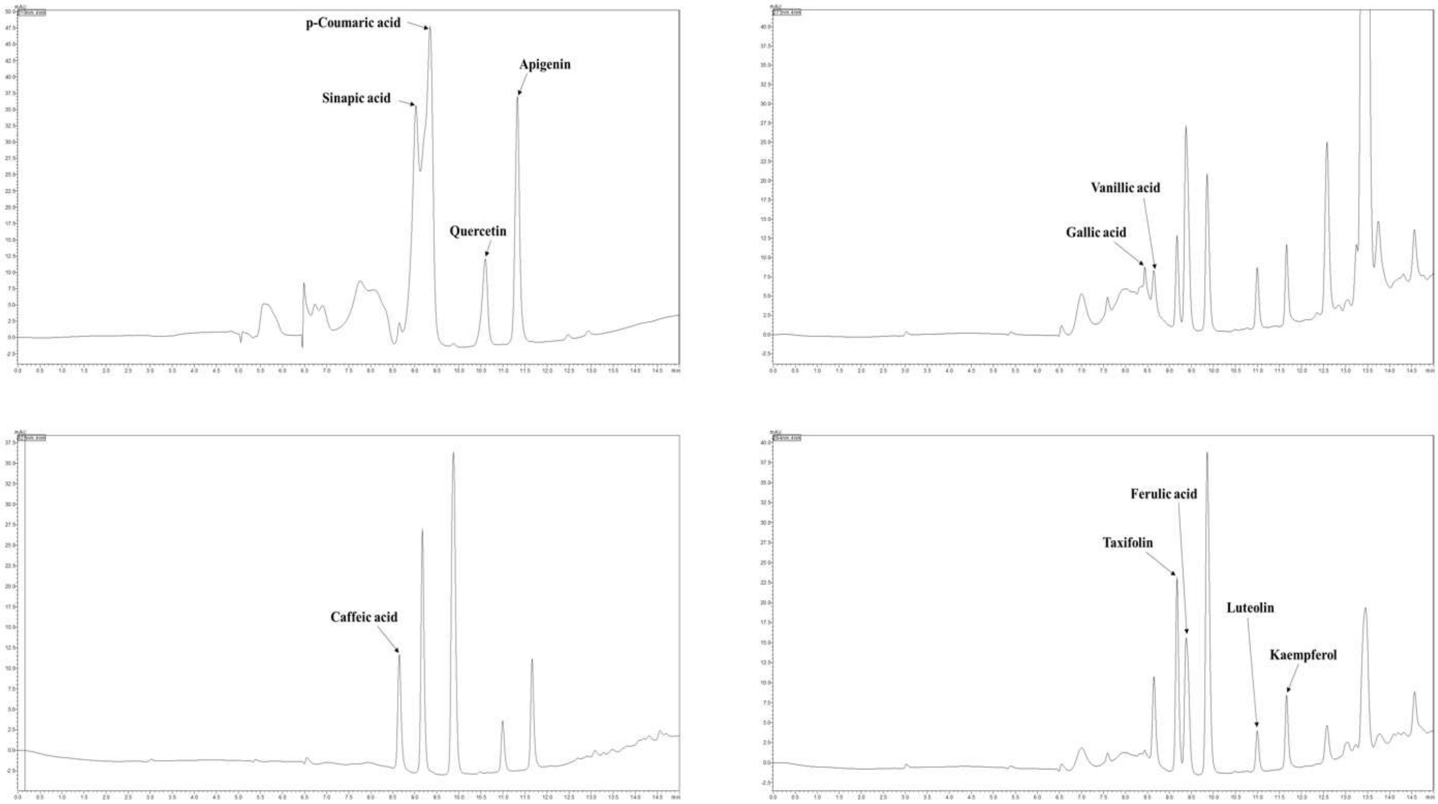

Supplementary Figure S1. An example of chromatographic separation of analytes in the sample (with peaks of each phenolic compound).

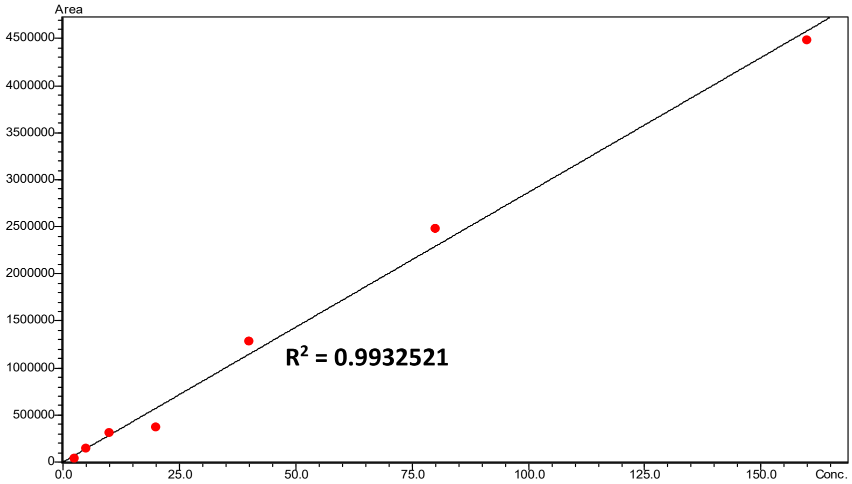
Area
4500000
4000000
3500000
3000000
2500000
2000000
1500000
1000000
500000
0
0.0
25.0
50.0
75.0
100.0
125.0
150.0
Conc.
R2 = 0.9932521

A

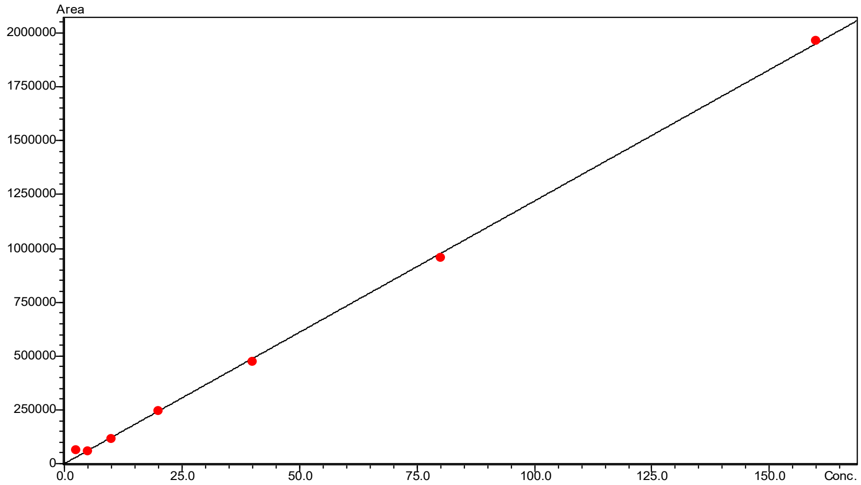
Area
2000000
1750000
1500000
1250000
1000000
750000
500000
250000
0
0.0
25.0
50.0
75.0
100.0
125.0
150.0
Conc.

B

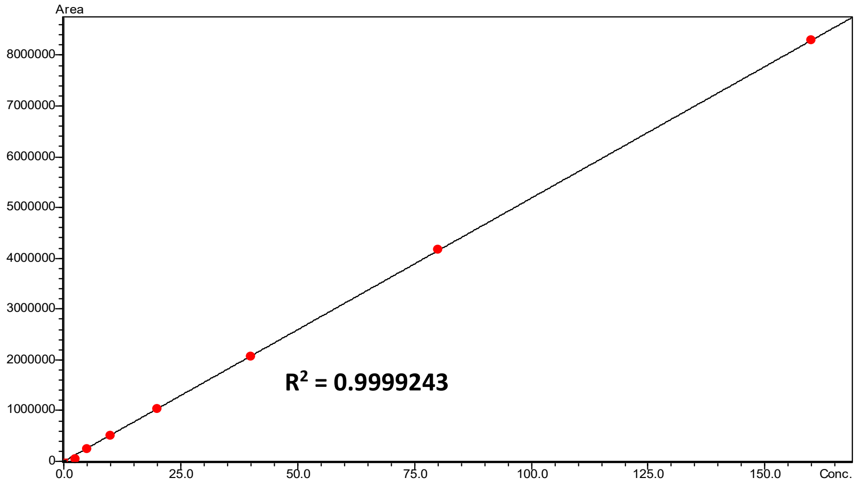
Area
8000000
7000000
6000000
5000000
4000000
3000000
2000000
1000000
0
0.0
25.0
50.0
75.0
100.0
125.0
150.0
Conc.
R2 = 0.9999243

C

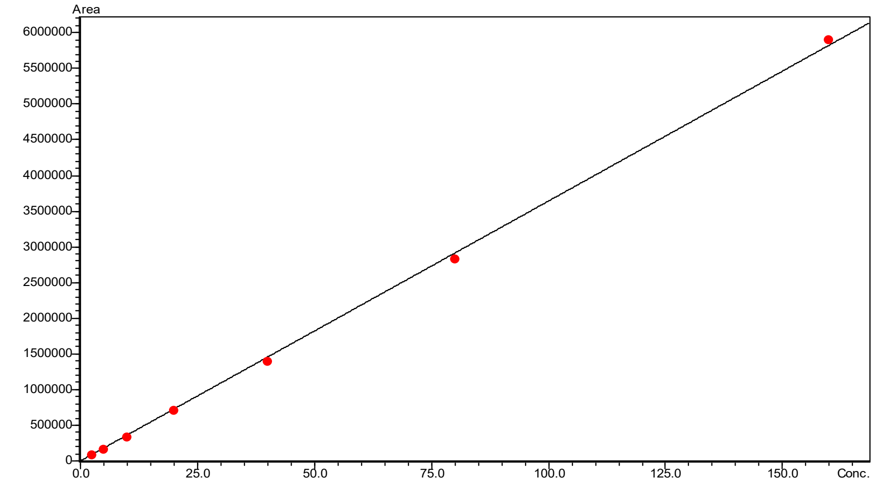
Area
6000000
5500000
5000000
4500000
4000000
3500000
3000000
2500000
2000000
1500000
1000000
500000
0
0.0
25.0
50.0
75.0
100.0
125.0
150.0
Conc.

D

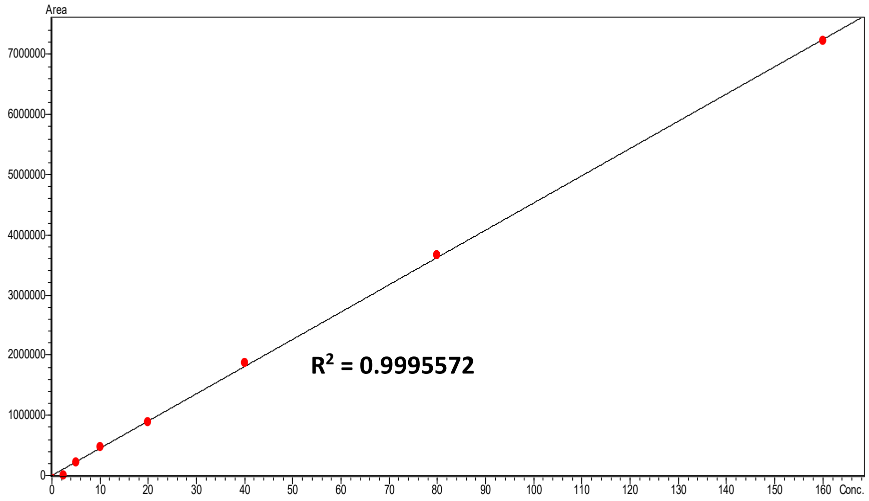
Area
7000000
6000000
5000000
4000000
3000000
2000000
1000000
0
0
10
20
30
40
50
60
70
80
90
100
110
120
130
140
150
160
Conc.
R2 = 0.9995572

E

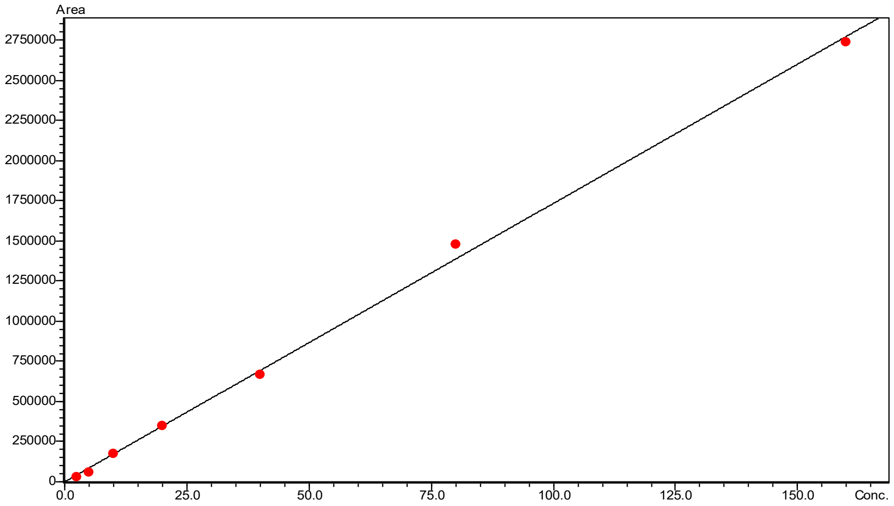
Area
2750000
2500000
2250000
2000000
1750000
1500000
1250000
1000000
750000
500000
250000
0
0.0
25.0
50.0
75.0
100.0
125.0
150.0
Conc.

F

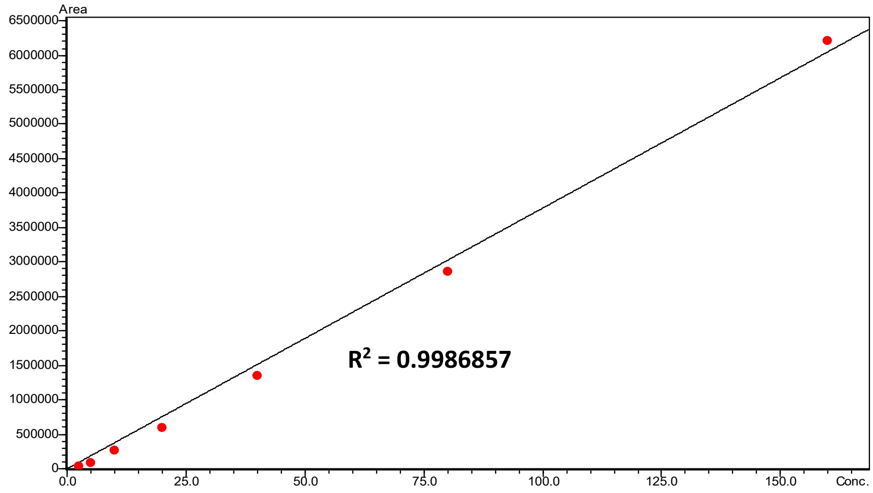
Area
6500000
6000000
5500000
5000000
4500000
4000000
3500000
3000000
2500000
2000000
1500000
1000000
500000
0
0.0
25.0
50.0
75.0
100.0
125.0
150.0
Conc.
R² = 0.9986857

G

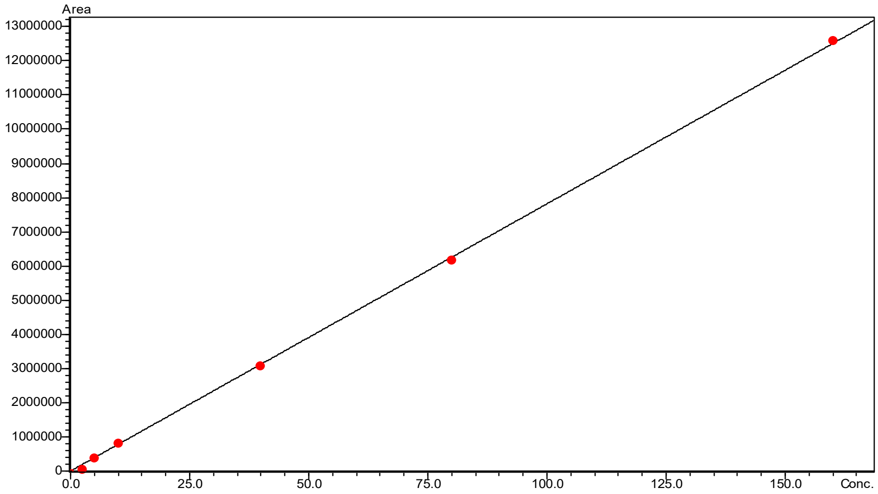
Area
13000000
12000000
11000000
10000000
9000000
8000000
7000000
6000000
5000000
4000000
3000000
2000000
1000000
0
0.0
25.0
50.0
75.0
100.0
125.0
150.0
Conc.

H

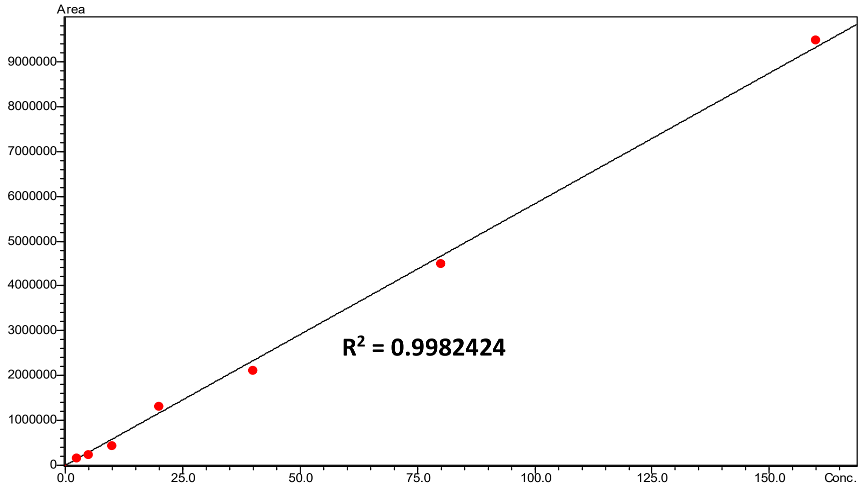
Area
9000000
8000000
7000000
6000000
5000000
4000000
3000000
2000000
1000000
0
0.0
25.0
50.0
75.0
100.0
125.0
150.0
Conc.
R² = 0.9982424

I

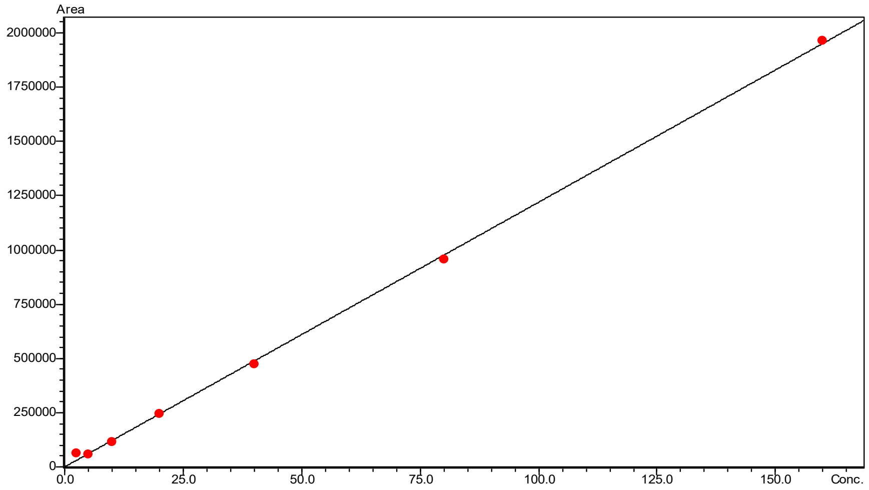

J

Supplementary Figure S2. Standard calibration curves and R^2 values for (A) apigenin, (B) kaempferol, (C) luteolin, (D) quercetin, (E) taxifolin, (F) caffeic acid, (G) ferulic acid, (G) gallic acid, (H) p-coumaric acid, (I) sinapic acid and (J) vanillic acid.
